# Pulsed Field Ablation in a Rheumatic Heart Disease Patient With Mechanical Mitral and Tricuspid Valves

**DOI:** 10.7759/cureus.94375

**Published:** 2025-10-12

**Authors:** Natee Deepan, Jervin Bisquera, Travis Fernandez, Alison Poppe, Pattara Rattanawong

**Affiliations:** 1 Biochemistry, Faculty of Medicine, Chulalongkorn University, Bangkok, THA; 2 Cardiology, Pali Momi Medical Center, Hawaii Pacific Health, Honolulu, USA; 3 Electrophysiology, Johnson and Johnson MedTech, Honolulu, USA

**Keywords:** atrial fib ablation, mechanical heart valves, pulsed-field ablation, rheumatic valvular disease, surgical replacement of valve

## Abstract

Pulsed field ablation (PFA) is a novel nonthermal modality for atrial fibrillation (AF) in patients with mechanical valves. We report the first successful PFA in a 44-year-old woman with both mechanical mitral and tricuspid valve replacements and a prior surgical maze procedure. Intracardiac echocardiography (ICE) and fluoroscopy-guided catheter navigation, avoiding prosthetic structures. Anatomical and voltage mapping revealed reconnection of prior surgical lines. PFA achieved isolation of the posterior wall, right pulmonary veins, inter-caval line, and cavotricuspid isthmus without complications. This case demonstrates the feasibility and safety of PFA in complex structural heart disease with dual mechanical valves.

## Introduction

Atrial fibrillation (AF) is highly prevalent among patients with mechanical prosthetic valves, occurring in approximately one-quarter of patients during hospitalization and affecting up to 30% after discharge following valve replacement surgery [1]. Management of AF in this population remains challenging: warfarin is mandatory for anticoagulation, rhythm-control drug options are limited, and catheter ablation--though feasible--is technically demanding due to altered atrial anatomy and prosthesis-related procedural risks [2]. Catheter or surgical ablation can be considered for rhythm control; however, in patients with mechanical valves, radiofrequency ablation is complicated by difficult catheter manipulation and potential entrapment or leaflet damage [3], whereas experience with cryoballoon ablation is extremely limited, with only isolated case reports and no systematic data available [4].

Pulsed field ablation (PFA), a nonthermal ablative technique [5], is a novel alternative for atrial fibrillation (AF) management in patients with mechanical prosthetic valves. In theory, PFA in patients with mechanical prosthetic valves raises concerns about potential field distortion, arcing near metallic components, catheter entrapment, and incomplete lesion formation due to prosthetic shielding [6]. We present the first reported case of PFA for persistent AF and atrial tachycardia in a patient with both mechanical mitral replacement (MVR) and mechanical tricuspid valve replacement (TVR). This case highlights the feasibility and safety of PFA in patients with mechanical MVR and TVR. 

## Case presentation

We present the successful case of PFA for AF in a patient with both mechanical MVR and TVR. A 44-year-old Pacific Islander woman with a 20-year history of rheumatic heart disease developed severe mitral stenosis and tricuspid regurgitation, and ultimately underwent elective MVR and TVR, with a 27- and 31-mm mechanical valve (CarboMedics Inc., Austin, TX). Due to the history of persistent atrial fibrillation, the surgery also included bipolar radiofrequency pulmonary veins (PVs) isolation (left maze), bipolar radiofrequency inter-caval line ablation (right maze), and surgical left atrial appendage excision. The surgery had been completed three years before the current presentation.

**Figure 1:**
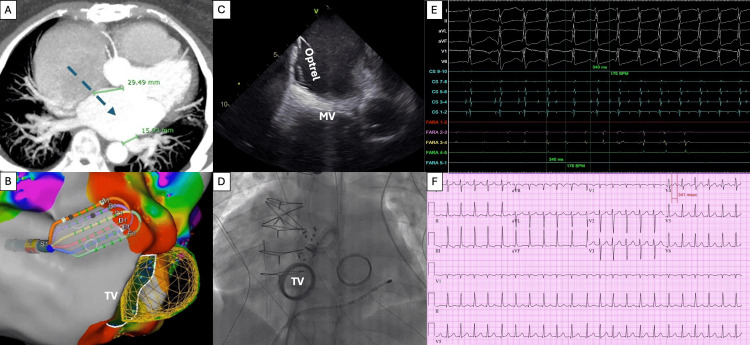
Pre-procedural and intra-procedural evaluation. (A) Pre-procedural computed tomography scan to evaluate pulmonary vein anatomy, left atrial appendage thrombus, and transseptal access planning. (B) The Optrell™ catheter was used, and an anatomical and voltage map was carefully obtained, avoiding the tricuspid mechanical valve, guided by (C) ICE and (D) fluoroscopy. (E) The 12-lead ECG of clinical atrial tachycardia. (F) The clinical atrial tachycardia was induced during the procedure. ICE: intracardiac echocardiography.

Since the surgery, the patient has had frequent hospital visits due to persistent AF and atrial tachycardia with rapid ventricular response, which required multiple cardioversions. Echocardiography revealed a new reduced left ventricular ejection fraction of 35-40%, left atrial enlargement (left atrial volume index: 38.9 mL/m²), and severe right atrial enlargement (right atrial area: 44.4 cm²). She was initially treated with metoprolol and amiodarone, but later discontinued due to symptomatic bradycardia. The PFA was considered. The patient underwent a pre-procedural computed tomography scan to evaluate the PV anatomy, left atrial appendage thrombus, and planning for transseptal access (Figure 1A). 

The procedure was conducted under general anesthesia. A decapolar coronary sinus catheter, intracardiac echocardiography (ICE), and a Faradrive™ steerable sheath (Boston Scientific Corporation, Marlborough, MA) were used. The patient was on therapeutic warfarin, and intravenous heparin was administered before transseptal puncture, targeting an activated clotting time above 400 seconds.

**Figure 2:**
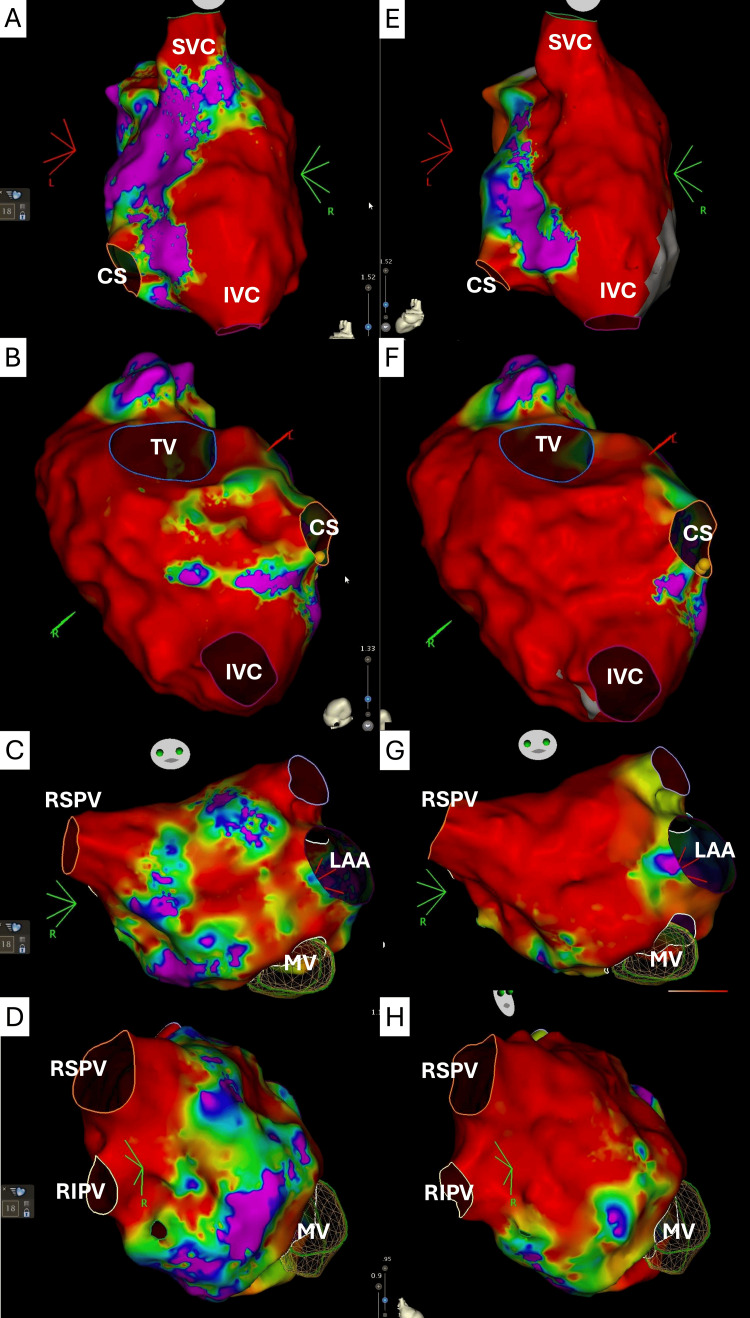
Electrophysiological findings and ablation outcomes. Fractionated electrograms with reconnection of (A) the inter-caval line from prior maze surgery, (B) CTI line area, (C) anterior wall, roof area, and (D) septal area near the septal border of the right pulmonary veins. Following ablation, a voltage map demonstrated (E) blocked inter-caval line, (F) blocked cavotricuspid isthmus line, (G) anterior mitral line, and (H) wide antral right pulmonary veins isolation. CS: coronary sinus; IVC: inferior vena cava; LAA: left atrial appendage; MV: mitral valve; SVC: superior vena cava; TV: tricuspid valve; RIPV: right inferior pulmonary vein; RSPV: right superior pulmonary vein; CTI: cavotricuspid isthmus.

The Carto 3™ system was used (Biosense Webster Inc., Irvine, CA). 3D CT integration into the mapping system provided accurate anatomic alignment, with valve position matching well to the electroanatomical geometry. ICE imaging helped delineate the mechanical tricuspid valve. The Optrell™ catheter (Biosense Webster Inc., Irvine, CA) was used, and an anatomical and voltage map was carefully obtained (Figure 1B), avoiding the tricuspid mechanical valve, guided by ICE (Figure 1C) and fluoroscopy (Figure 1D). The sinus node was mapped in anticipation of possible ablation near the superior vena cava (SVC), and the His bundle was tagged (Figures 2A, 2E). Fractionated electrograms with reconnection of the inter-caval line from prior maze surgery and cavotricuspid isthmus (CTI) line area were identified (Figure 2B).

A single transseptal puncture was performed using the VersaCross Connect™ (Boston Scientific Corporation, Marlborough, MA) and VersaCross RF Wire™ (Boston Scientific Corporation, Marlborough, MA). An anatomical and voltage map was created, avoiding the mitral mechanical valve. Both left and right PVs were found isolated. The floor line was found intact, and the posterior wall was found partially isolated. The roof line was found reconnected. Fractionated electrograms were identified at the anterior wall, roof area, and septal area near the septal border of the right PVs (Figures 2C, 2D).

The Farawave™ catheter (Boston Scientific Corporation, Marlborough, MA) was positioned at the roof line, anterior mitral line, and septal border of the right PVs. Biphasic pulse train applications were delivered in flower configurations, with a total of two, 14, and 10 lesions applied at the roof line, anterior mitral line, and septal border of the right PVs, respectively. The Farawave™ catheter was carefully positioned near the mitral mechanical valve under ICE and fluoroscopy guidance (Figure 1D).

For right atrial ablation, the Farawave™ catheter was maneuvered to the CTI region. Intravenous nitroglycerine was administered to prevent vasospasm, and phenylephrine was given to maintain blood pressure. A total of 14 ablation lesions were delivered in a flower shape along the CTI line. During cavotricuspid isthmus ablation with the Farawave™ catheter, the device should be maintained on the atrial aspect of the tricuspid annulus with minimal torque and without deep advancement toward the valve leaflets or septal annulus to avoid leaflet interaction and AV nodal injury [7].

In the SVC, a brief episode of atrial tachycardia (Figure 1E) was induced with Farawave™ catheter manipulation at the inferior border of the SVC, initiated by a premature atrial complex at Farawave™ 3,4, near the reconnected area of the inter-caval line with the same atrial tachycardia cycle length (340 ms) to clinical atrial tachycardia (Figure 1F). This terminated spontaneously. Six ablation lesions in a flower shape were created along the inferior SVC border to retouch the inter-caval line (Figure 2A) from the previous right atrial maze surgery. Post-ablation voltage maps confirmed inter-caval and CTI and line block (Figures 2E, 2F) compared to the pre-ablation map (Figures 2A, 2B). AF was not observed throughout the procedure. 

Following ablation, a voltage map demonstrated posterior wall isolation, anterior mitral line, and wide antral right PVs isolation (Figures 2G, 2H). Anterior mitral line block was confirmed with differential pacing. Atrial burst pacing and double extra stimuli, without isoproterenol, failed to induce atrial tachycardias or atrial fibrillation. She was discharged home with one and three months scheduled follow-up, during which no atrial arrhythmia was documented.

## Discussion

Radiofrequency ablation or cryoablation poses risks of thermal and mechanical injury to adjacent structures, especially in patients with prosthetic valves. PFA has recently gained attention as a nonthermal modality that selectively targets myocardial cells while sparing surrounding tissues, making it an attractive option for complex patients, including those with mechanical valves.

We present the first use of PFA in a patient with both MVR and TVR. Prior studies have demonstrated the safety and efficacy of PFA in patients with mechanical MVR and repaired tricuspid valves [8] or mechanical MVR and aortic valve replacement [6], but the feasibility of PFA in the setting of both MVR and TVR has not been well established.

In this case, ICE and fluoroscopy were used to facilitate catheter navigation and avoid mechanical valve structures. Unlike traditional mapping catheters such as PentaRay or OctaRay, which can become entrapped in prosthetic valves [9-11], we used the Optrell catheter to enhance safety. Additionally, left atrial computed tomography scan imaging was incorporated into procedural planning, allowing for better visualization of PVs anatomy and ensuring precise energy delivery.

Another consideration is the effect of mechanical prostheses on lesion formation. Metallic valves may alter electric field propagation and affect ablation efficacy, particularly in the tricuspid annulus, where the valve structure could theoretically act as a conductor. However, in this case, lesion formation was not compromised. Additionally, reconnection of the inter-caval line from prior maze surgery was targeted with PFA, demonstrating the feasibility of re-intervening on prior ablation lesions.

PFA represents a promising and minimally invasive approach for AF ablation in patients with MVR and TVR. This case highlights its feasibility and safety, demonstrating successful rhythm control with PFA in a patient with mechanical MVR and TVR without procedural complications. The use of PFA in this context may offer unique advantages, including the avoidance of thermal injury and reduced risk of catheter entrapment within the mechanical valve apparatus. These are meaningful safety considerations in patients with prosthetic valve hardware, where thermal energy sources may pose added risk. Further research is needed to validate PFA's efficacy and long-term outcomes in this patient population.

This case underscores the potential for PFA to be a safe and effective alternative for AF ablation in patients with multiple mechanical valves. However, additional studies are needed to confirm its long-term efficacy and safety in this complex subset of patients.

## Conclusions

PFA represents a promising and minimally invasive approach for AF ablation in patients with MVR and TVR. This case highlights its feasibility and safety, demonstrating successful rhythm control with PFA in a patient with mechanical MVR and TVR without procedural complications. Further research is needed to validate PFA's efficacy and long-term outcomes in this patient population.
